# Robotic-assisted compared to conventional laparoscopic surgery for colorectal endometriosis: perioperative outcomes in the context of #Enzian-defined anatomical complexity

**DOI:** 10.1007/s11701-026-03634-9

**Published:** 2026-07-20

**Authors:** Harald Krentel, L. Burla, P. Tanovska, P. Klein, C. Hoche, M. Constantin, M. S. de Wilde, R. Devassy, J. Keckstein, I. Vlachodimitris, P. Sklavounos, D. Andrikos

**Affiliations:** 1 Department of Gynecology and Obstetrics, Klinikum Aschaffenburg-Alzenau, Aschaffenburg, Germany; 2 Department of Gynecology, University Hospital Zurich, Zurich, Switzerland; 3 Clinic of Gynecology, Obstetrics and Gynecological Oncology, University Hospital for Gynecology, Pius-Hospital Oldenburg, Oldenburg, Germany; 4 Dubai London Clinic, Dubai, UAE; 5 Endometriosis Clinic Dres. Keckstein, Villach, Austria; 6 Center for Endometriosis Care, EndoAthens, Athens, Greece; 7 Andrikos Clinic, Womens Health Experts, Thessaloniki, Greece; 8 Klinikum Aschaffenburg-Alzenau, Aschaffenburg, Germany

**Keywords:** Colorectal endometriosis, Robotic surgery, Laparoscopy, #Enzian classification, Deep infiltrating endometriosis

## Abstract

The objective of this work was to compare the perioperative safety and operative efficacy of robotic-assisted surgery (RAS) versus conventional laparoscopy surgery (CLS) in women with symptomatic colorectal endometriosis, with particular focus on to the anatomical disease complexity as defined by the #Enzian system. We retrospectively reviewed 160 consecutive cases operated for colorectal endometriosis at a single referral center between 2022 and 2025. Patients were managed by RAS (*n* = 59) or CLS (*n* = 101) in a non-randomized, sequential setting, with CLS performed prior to the implementation of RAS at our center. Intraoperative variables (operative time, blood loss, number of trocars, conversion rate) and perioperative outcomes (length of stay, transfusion, intensive-care admission, complications, reoperation, readmission, anastomotic leakage, and C-reactive protein serum levels on postoperative days 1–3 were analyzed. Anatomical disease extent was assessed using the #Enzian classification, including compartments P, O, T, A, B, C and F-compartments (FA, FB, FI, FU, F-nerves, F-diaphragm). Continuous variables were compared using the Mann–Whitney U test and categorical variables using Fisher’s exact test, with *p* < 0.05 considered statistically significant. Complete excision of colorectal endometriosis and additional lesions was achieved with either route in all cases, with a very low overall rate of anastomotic leakage (2/160; 1.3%). Anatomical complexity was significantly higher in the RAS group, with more frequent peritubal/periovarian adhesions (T) (*p* = 0.002), advanced rectal (C) lesions (*p* = 0.003), and increased involvement of the bladder (FB) (8.5% vs. 0.0%, *p* = 0.006), ureter (FU) (11.9% vs. 2.0%, *p* = 0.013), and diaphragm (10.2% vs. 0.0%, *p* = 0.002). Despite this, median operative time was comparable between groups (225 [150–292] vs. 201 [146–251] minutes, *p* = 0.188). Length of hospital stay was significantly shorter after RAS (5 [4–6] vs. 6 [4–7] days, *p* = 0.008). Overall complication rates were similar (10.2% vs. 12.9%, *p* = 0.80), and the reoperation rate was numerically lower in the RAS group (1.7% vs. 6.9%, *p* = 0.26). RAS and CLS are both safe and effective surgical approaches for excision of symptomatic colorectal endometriosis. Despite being used in anatomically more complex cases, RAS achieved perioperative outcomes comparable to CLS, with a shorter length of hospital stay and a numerically lower reoperation rate. These findings underscore the importance of accounting for anatomical complexity and disease extent when comparing outcomes in deep endometriosis surgery. Prospective risk-adjusted or propensity score–matched studies are warranted to confirm these results.

**What is new?** RAS achieves comparable perioperative outcomes to CLS in colorectal endometriosis and is associated to shorter hospitalization, despite being preferentially used in anatomically more complex cases defined by the #Enzian classification.

## Introduction

Deep endometriosis (DE) affects approximately 20% of women with endometriosis and may involve the rectovaginal septum, parametria, urinary tract, bowel, pelvic nerves and the diaphragm. Colorectal involvement occurs in 8–12% of patients with DE and is associated with symptoms such as dyschezia, hematochezia, dyspareunia, defecatory dysfunction, bloating, and infertility, resulting in a significant impairment of quality of life [1, 2]. When medical treatment fails, complete surgical excision of symptomatic deep endometriosis is indicated. Three surgical techniques are typically described for rectal endometriosis: rectal shaving or complete mucosa-sparing nodular resection, discoid excision, and segmental resection with anastomosis [3].

Conventional laparoscopy (CLS) has been the standard minimally invasive approach for colorectal endometriosis for more than two decades and achieves excellent outcomes when performed by experienced multidisciplinary teams [4]. Robotic-assisted surgery (RAS) has been progressively introduced in the management of DE over the last years, offering optimized vision, greater range of motion of instruments, tremor filtering and superior ergonomics [5]. These technical features are considered potentially beneficial in anatomically demanding retroperitoneal regions of the pelvis such as the deep parametrium, presacral spaces, pararectal spaces, the diaphragm and in complex multidisciplinary approaches. Previous studies suggest that surgeons preferentially use RAS in complex and multidisciplinary cases of deep endometriosis [6].

However, evidence comparing RAS and CLS for deep endometriosis remains heterogeneous. While Soto et al. reported no differences in perioperative outcomes comparing robotic-assisted surgery and conventional laparoscopy [7], meta-analyses have shown a trend towards prolonged operative times for RAS, with comparable or lower complication rates and very low conversion rates [8, 9]. Importantly, most published series do not adjust for the anatomical extent of the disease, which is known to be a major determinant of operative complexity, surgical time and morbidity. The #Enzian classification provides a reproducible topographic description of DE, covering the peritoneum (P), the ovaries (O), the tubo-ovarian unit (T), the rectovaginal space and vagina (A), uterosacral ligaments and parametria (B), the rectum (C) and the F-compartments (FA = adenomyosis, FB = bladder, FI = non-rectum bowel, FU = ureter, and other localizations like F(nerves) or F(diaphragm) [10].

The aim of this study was to analyze the safety and the perioperative outcomes of RAS and CLS in the surgical management of colorectal endometriosis in a consecutive single-center cohort, while explicitly accounting for anatomical disease complexity using the #Enzian classification.

## Materials and methods

### Study design

This retrospective single-center cohort study compared outcomes of RAS and CLS in the surgical management of colorectal endometriosis. The analysis included all patients who underwent robotic-assisted surgery or conventional laparoscopic surgery for rectal endometriosis based on preoperative transvaginal ultrasound examination at the Department of Obstetrics and Gynecology at Bethesda Hospital Duisburg. Only patients with histologically confirmed rectal endometriosis were included in the final analysis. Treatment allocation was not randomized. CLS procedures were performed before the implementation of the robotic program. After implementation of the robotic program, RAS was performed using the da Vinci X multi-port system (Intuitive Surgical, Sunnyvale, CA, USA). The surgical objective in all cases was complete macroscopic excision of endometriotic lesions. Patients were excluded if perioperative data were incomplete or if surgery was performed for deep endometriosis without rectal involvement. The perioperative management followed a standard protocol for rectal endometriosis surgery and patients were discharged based on clinical course, postoperative well-being, infection parameters, and return of bowel function.

### Surgical management

All procedures in both cohorts were carried out by the same experienced surgeon and the same multidisciplinary team in a referral center for colorectal endometriosis surgery. All cases were managed following an interdisciplinary standard procedure including the team from colorectal surgery and/or urology. Perioperative management protocols did not change during the study period. Disease extent was classified intraoperatively according to the #Enzian classification, including F-compartment involvement. Depending on presurgical imaging and intraoperative findings, rectal endometriosis was managed either by complete mucosa-sparing nodular resection (CNR) or segmental rectal resection (SRR), as previously reported by our group. Resection of the appendix, sigmoid, coecum or small bowel were performed when indicated by FI-compartment disease. In cases of bladder (FB) or ureteral (FU) involvement, partial cystectomy, ureterolysis and, if necessary, ureteral re-implantation were performed. Diaphragmatic lesions were treated by laparoscopic or robotic excision.

#### Conventional laparoscopy (CLS)

CLS was performed using a standard four- or five-trocar configuration. The surgical steps followed the standardized anatomical approach for deep endometriosis including mobilization of the sigmoid, opening of the retroperitoneal and pararectal spaces, nerve-sparing parametrial dissection including ureterolysis and complete excision of deep disease.

#### Robotic-assisted laparoscopy (RAS)

RAS was performed using a standardized port configuration with three or four robotic arms and one assistant trocar. The robotic platform was used throughout the procedure, including colorectal and urological steps when required. Surgical principles were identical to those used in CLS, including standardized anatomical dissection and complete macroscopic excision of deep disease.

### Ethical considerations

The study was conducted in accordance with the principles of the Declaration of Helsinki and the recommendations of the Committee on Publication Ethics (COPE). All included participants granted written patient consent regarding the anonymous use of their medical data for research purposes.

### Ethics committee approval

The study protocol was reviewed and approved by the independent Ethical Review Board of Bethesda Hospital Duisburg and Evangelic Hospital Niederrhein under the number IR-15-2025 in August 2025.

### Data collection

Electronic medical records of all included patients were retrospectively reviewed. The following variables were extracted: surgical approach (RAS/CLS), skin-to-skin operative time and console time (for RAS cases), length of stay, intraoperative blood loss, number of trocars, conversion to open surgery, transfusion requirement, intensive-care admission, any complication, reoperation, readmission, anastomotic/suture leakage, mortality, CRP values on postoperative days 1–3, and the #Enzian classification of disease extent. The F-compartments were coded as binary variables (0 = not involved, 1 = involved) for FA (adenomyosis), FB (bladder), FI with its four subsites (Appendix, Sigmoid, Cecum, Ileum), FU (ureter) left and right, F-Nerves and F-Diaphragm. Perioperative complications were reported according to the Clavien-Dindo classification.

###  Statistical analysis

Descriptive statistics were used to summarize baseline, intraoperative, perioperative, and anatomical variables. Continuous variables are expressed as median with interquartile range (IQR) and as mean ± standard deviation. Categorical variables are expressed as absolute frequencies and percentages. Between-group comparisons were performed using the Mann-Whitney U test for continuous variables and Fisher’s exact test for categorical variables. As a proxy for anatomical complexity, the number of involved #Enzian compartments was calculated for each patient by assigning one point for each involved compartment among P, O, T, A, B, C, FA, FB, FI, FU, F-nerves, and F-diaphragm. For P, O, T, A, B, and C compartments, any grade of involvement [1–3] was counted as involved; F-compartments were counted as involved when coded positive.We deliberately did not compute a cumulative numerical #Enzian score, as the #Enzian system is descriptive and topographical and is not intended to be used as an interval-scale value. A two-sided p value < 0.050 was considered statistically significant. Given the retrospective design and non-randomized sequential treatment allocation, all comparative analyses were considered exploratory. Statistical analysis were performed using Python 3 with pandas and NumPy.

## Results

### General characteristics

A total of 160 consecutive patients undergoing surgery for histologically confirmed colorectal endometriosis were included. Of these, 59 (36.9%) were managed by robotic-assisted laparoscopy and 101 (63.1%) by conventional laparoscopy. In the RAS group, 47.5% of cases were managed by complete mucosa-sparing nodular resection (CNR) and 52.5% by segmental rectal resection (SRR) with anastomosis. In the CLS group the distribution was similar with 40.6% of CNR and 59.4% of SRR. Overall cohort characteristics and unadjusted comparisons between the RAS and CLS groups are summarized in Table 1.

**Table 1:** General characteristics of the whole cohort (*n* = 160) and comparison between RAS and CLS

Variable	Whole cohort (*n* = 160)	RAS (*n* = 59)	CLS (*n* = 101)
Skin-to-skin operative time, median [IQR], minutes	205 [150–273]	225 [150–292]	201 [146–251]
Skin-to-skin operative time, mean ± SD, minutes	213.8 ± 81.7	225.1 ± 83.0	207.1 ± 80.8
Length of hospital stay, median [IQR], days	5 [4–7]	5 [4–6]	6 [4–7]
Length of hospital stay, mean ± SD, days	5.5 ± 2.0	5.1 ± 2.0	5.7 ± 2.0
Estimated blood loss, median [IQR], mL	100 [100–150]	100 [100–138]	100 [100–150]
Number of trocars, median [IQR]	5 [4–5]	4 [4–5]	5 [4–5]
Number of involved #Enzian compartments, median [IQR]	5 [4–7]	6 [5–7]	5 [4–6]
Any complication, n (%)	19 (11.9)	6 (10.2)	13 (12.9)
Conversion to laparotomy, n (%)	1 (0.6)	1 (1.7)	0 (0.0)
Reoperation, n (%)	8 (5.0)	1 (1.7)	7 (6.9)
Readmission, n (%)	10 (6.3)	4 (6.8)	6 (5.9)
Blood transfusion, n (%)	1 (0.6)	1 (1.7)	0 (0.0)
Intensive-care admission, n (%)	1 (0.6)	1 (1.7)	0 (0.0)
Anastomotic or suture leakage, n (%)	2 (1.3)	0 (0.0)	2 (2.0)
Mortality, n (%)	0 (0.0)	0 (0.0)	0 (0.0)

### RAS versus CLS: intraoperative and perioperative outcomes

Median skin-to-skin operative time was 225 min (IQR 150–292) in the RAS group and 201 min (IQR 146–251) in the CLS group (*p* = 0,188). The mean console time in the RAS group was 150.3 ± 69.4 min. The median number of trocars was lower in the RAS group than in the CLS group (4 [4–5] vs. 5 [4–5], *p* = 0.004).

Estimated intraoperative blood loss was comparable between both groups (median 100 mL in each group, *p* = 0.075). Conversion to open surgery was rare and occurred exclusively in the RAS group (1/59, 1.7% vs. 0/101, 0.0%; *p* = 0.37).

Length of stay was significantly shorter after RAS than after CLS (median 5 [4–6] vs. 6 [4–7] days, *p* = 0.008). Postoperative CRP levels were higher after RAS on postoperative day 2 (median 4.32 vs. 2.80 mg/dL, *p* = 0.035) and day 3 (median 3.25 vs. 2.01 mg/dL, *p* = 0.007). Despite higher CRP levels, overall complication rates were similar (10.2% vs. 12.9%, *p* = 0.80) and reoperation did not differ significantly between groups.

The reoperation rate was numerically lower after RAS than after CLS (1.7% vs. 6.9%; *p* = 0.26), Readmission rates were comparable (6.8% vs. 5.9%, *p* = 1.00). Comparative intraoperative and perioperative outcomes are summarized in Table 2.

Each perioperative complication was classified according to the Clavien-Dindo grading system on the basis of the documented clinical management. The distribution of Clavien-Dindo grades is shown in Table 3. Overall complications occurred in 6 RAS patients (10.2%) and 13 CLS patients (12.9%). Severe complications (Clavien-Dindo ≥ IIIa) occurred in one RAS patient (1.7%) and seven CLS patients (6.9%). Anastomotic or suture leakage occurred in 2 patients of the CLS group (2.0%) and in no patient of the RAS group (*p* = 0.55). In both cases of leakage a temporary stoma formation was necessary. Blood transfusion (1/59, 1.7% vs. 0/101, 0.0%) and intensive-care admission (1/59, 1.7% vs. 0/101, 0.0%) were rare in both groups. Temporary voiding dysfunction was reported in 3 patients after conventional laparoscopic SRR. The difference in severe morbidity was not statistically significant (*p* = 0.26). No Clavien-Dindo grade IV or V complications occurred in either group.

**Table 2:** Intraoperative and perioperative outcomes according to surgical approach

Variable	RAS (*n* = 59)	CLS (*n* = 101)	*p*
Skin-to-skin operative time, median [IQR], minutes	225 [150–292]	201 [146–251]	0.188
Console time, mean ± SD, minutes	150.3 ± 69.4	—	—
Length of hospital stay, median [IQR], days	5 [4–6]	6 [4–7]	0.008
Estimated blood loss, median [IQR], mL	100 [100–138]	100 [100–150]	0.075
Number of trocars, median [IQR]	4 [4–5]	5 [4–5]	0.004
CRP postoperative day 1, median, mg/dL	3.21	2.33	0.063
CRP postoperative day 2, median, mg/dL	4.32	2.80	0.035
CRP postoperative day 3, median, mg/dL	3.25	2.01	0.007
Any complication, n (%)	6 (10.2)	13 (12.9)	0.80
Reoperation, n (%)	1 (1.7)	7 (6.9)	0.26
Conversion to laparotomy, n (%)	1 (1.7)	0 (0.0)	0.37
Readmission, n (%)	4 (6.8)	6 (5.9)	1.00
Anastomotic/suture leakage, n/N (%)	0/59 (0.0)	2/101 (2.0)	0.55

**Table 3:** Clavien-Dindo classification of perioperative complications according to surgical approach

Clavien-Dindo grade	RAS (*n* = 59)	CLS (*n* = 101)
Grade I	0 (0.0)	3 (3.0)
Grade II	5 (8.5)	3 (3.0)
Grade IIIa	0 (0.0)	1 (1.0)
Grade IIIb	1 (1.7)	6 (5.9)
Grade IVa	0 (0.0)	0 (0.0)
Grade IVb	0 (0.0)	0 (0.0)
Grade V	0 (0.0)	0 (0.0)
Any complication	6 (10.2)	13 (12.9)
Severe complication (grade ≥ IIIa)	1 (1.7)	7 (6.9)

### Anatomical complexity according to #Enzian classification

**Fig. 1:**
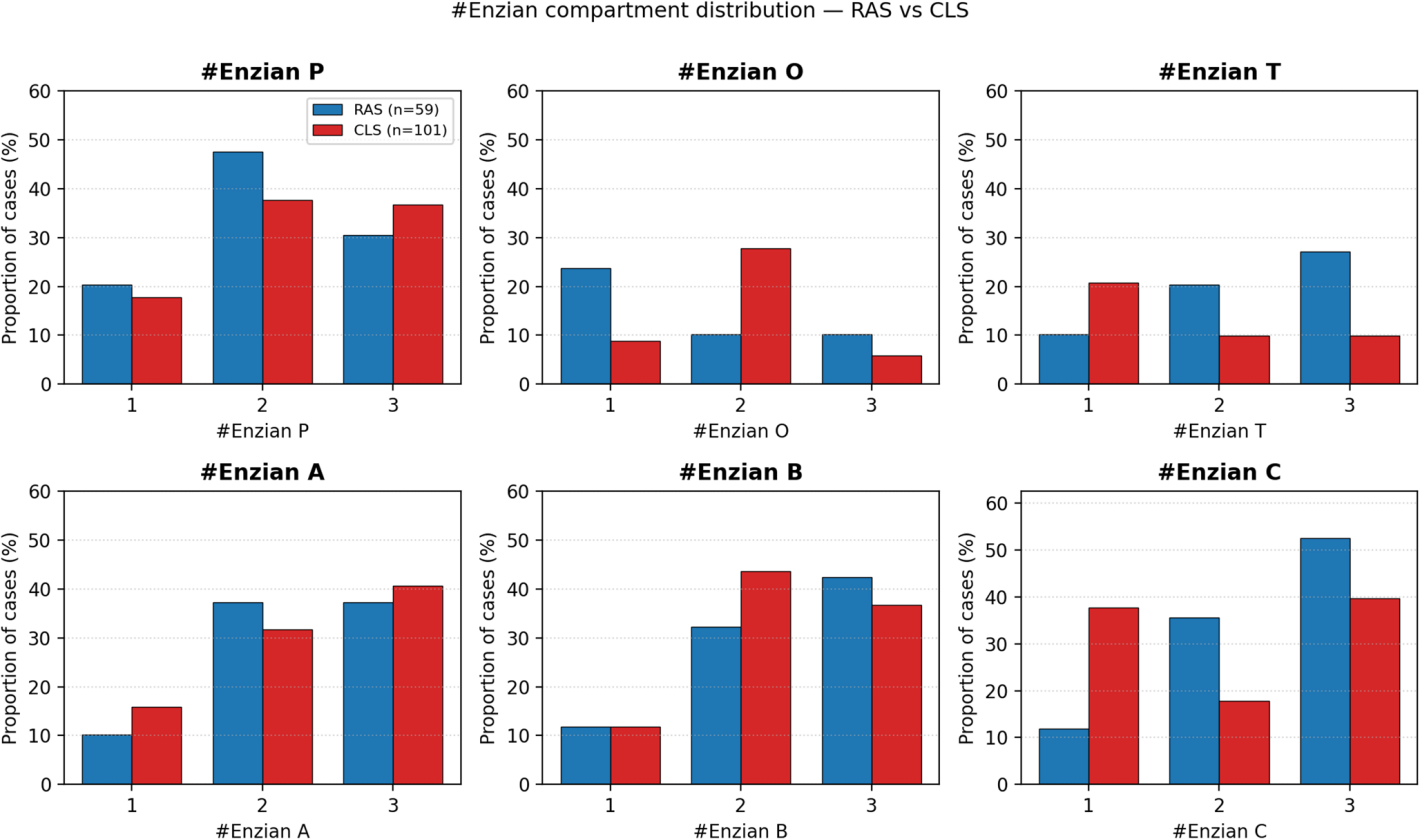
Percentage distribution of the #Enzian compartments (P, O, T, A, B, C) in the RAS and CLS groups. Higher-grade T- and C-involvement was more frequent in the RAS group

As a proxy for anatomical complexity, the number of involved #Enzian compartments (P, O, T, A, B, C and the F-compartments) was computed for each patient. It was significantly higher in the RAS group (median 6 [IQR 5–7] vs. 5 [IQR 4–6], *p* = 0.017). The compartment-by-compartment analysis showed that peritubal and periovarian adhesions (T) (*p* = 0.002) and rectal (C) (*p* = 0.003) disease was significantly more severe in the RAS group (Figs. 1 and 3). The distribution of P, O, A and B stages was comparable between the two groups.

### F-compartments

**Fig. 2:**
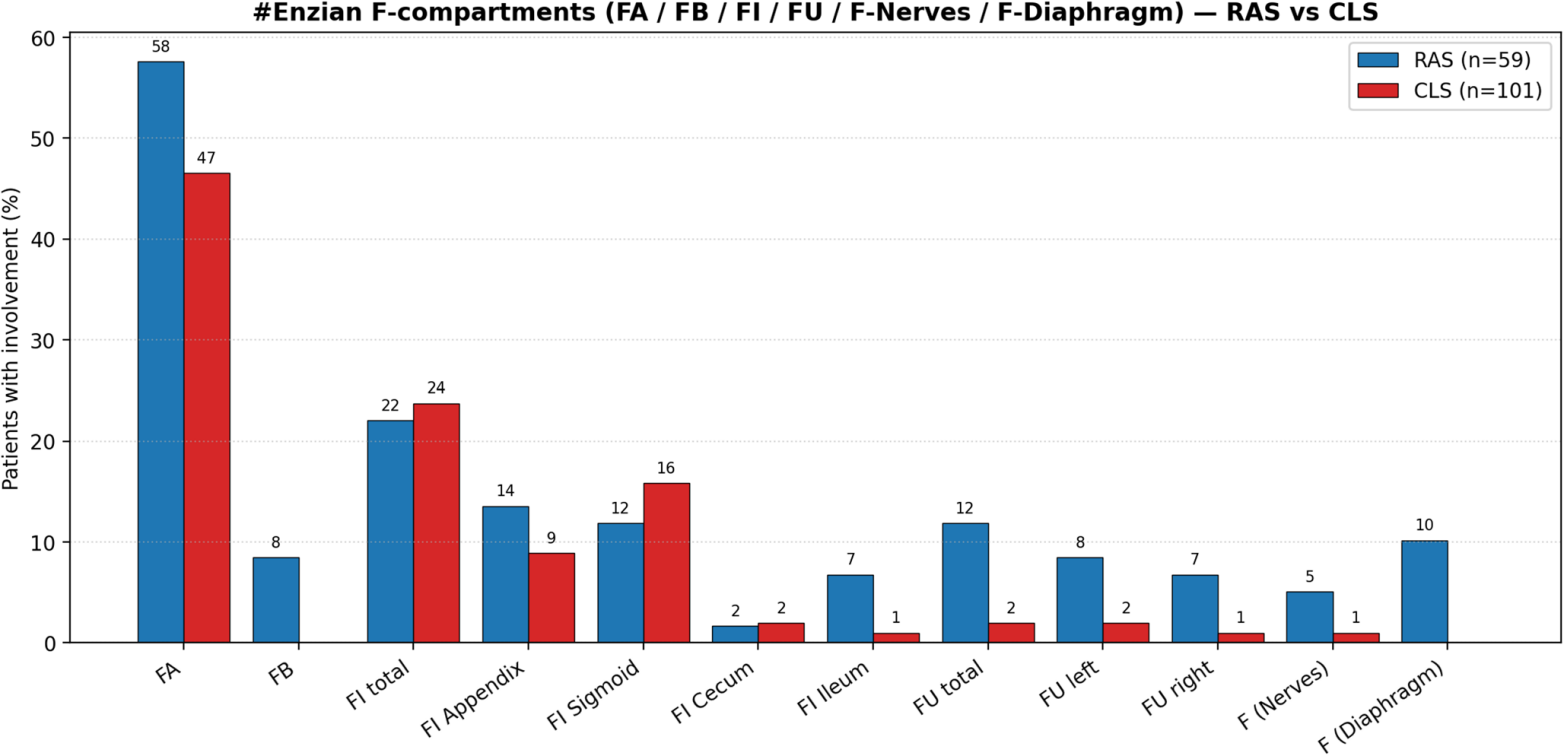
Rate of involvement of the #Enzian F-compartments (FA, FB, FI subsites, FU, F-Nerves, F-Diaphragm) in the RAS and CLS groups

**Fig. 3:**
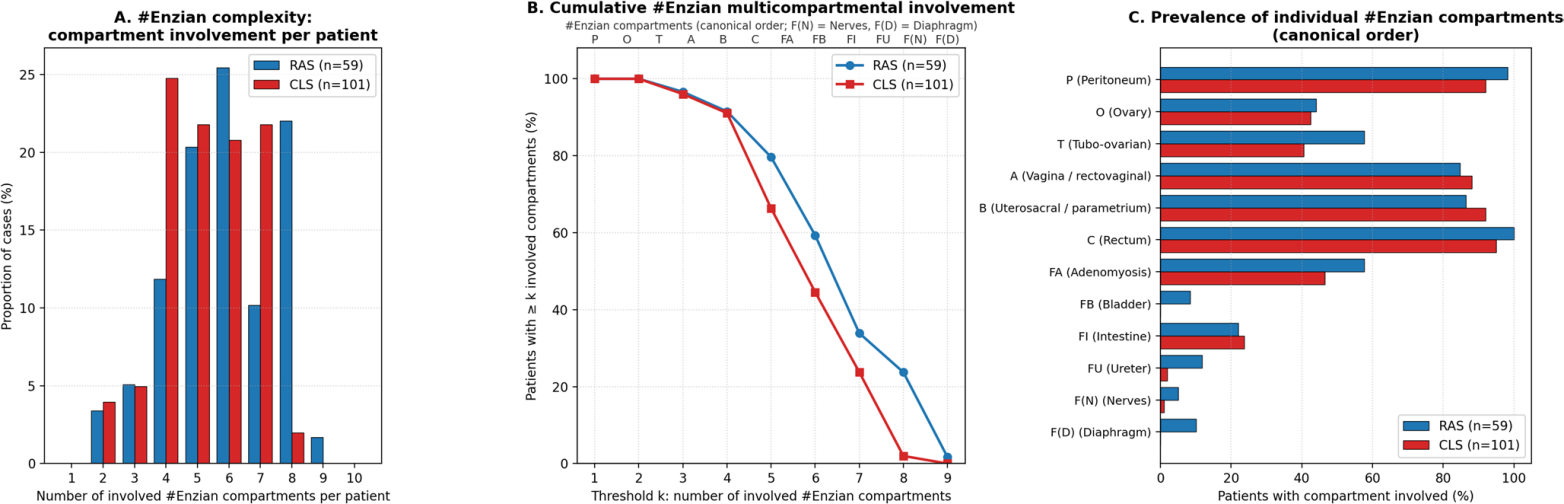
Anatomical complexity according to the #Enzian classification. Left: distribution of the number of involved #Enzian compartments per patient. Center: cumulative involvement regarding #Enzian compartments. Right: cumulative distribution of multicompartmental involvement (proportion of patients with ≥ k involved compartments) in the RAS and CLS groups. F(N)=Nerves; F(D)=Diaphragm

Binary analysis of the F-compartment involvement (0 = not involved, 1 = involved) revealed a significantly higher prevalence of extensive multicompartmental disease in the RAS group (Table 4; Fig. 2). The rate of FA (adenomyosis) was comparable (57.6% vs. 46.5%, *p* = 0.19). FB (bladder) involvement occurred exclusively in the RAS group (8.5% vs. 0.0%, *p* = 0.006). Overall FU involvement (ureter) was more frequent in the RAS group (11.9% vs. 2.0%, *p* = 0.013). Overall FI involvement (non-rectal bowel) did not differ significantly between groups (22.0% vs. 23.8%, *p* = 0.85), whereas ileal involvement was numerically more frequent in the RAS group (6.8% vs. 1.0%, *p* = 0.062). F-nerves involvement was observed in 5.1% of RAS cases and 1.0% of CLS cases (*p* = 0.14). Diaphragmatic endometriosis (F-Diaphragm) was observed exclusively in the RAS group (10.2% vs. 0.0%, *p* = 0.002). Figure 3 displays the distribution of anatomical complexity (number of involved #Enzian compartments) between the two groups.

**Table 4:** Distribution of #Enzian F-compartment involvement according to surgical approach

Compartment	RAS, *n*/*N* (%)	CLS, *n*/*N* (%)	*p*
FA	34/59 (57.6)	47/101 (46.5)	0.19
FB	5/59 (8.5)	0/101 (0.0)	0.006
FI overall (non-rectal bowel)	13/59 (22.0)	24/101 (23.8)	0.85
FI Appendix	8/59 (13.6)	9/101 (8.9)	0.43
FI Sigmoid colon	7/59 (11.9)	16/101 (15.8)	0.64
FI Coecum	1/59 (1.7)	2/101 (2.0)	1.00
FI Ileum	4/59 (6.8)	1/101 (1.0)	0.062
FU overall (ureter)	7/59 (11.9)	2/101 (2.0)	0.013
FU left	5/59 (8.5)	2/101 (2.0)	0.10
FU right	4/59 (6.8)	1/101 (1.0)	0.062
F Nerves	3/59 (5.1)	1/101 (1.0)	0.14
F Diaphragm	6/59 (10.2)	0/101 (0.0)	0.002

## Discussion

Symptomatic deep endometriosis of the rectum is considered a challenging scenario in benign gynecologic surgery and often requires a multidisciplinary approach combining advanced gynecological laparoscopy with colorectal surgical expertise. In this consecutive single-center cohort of 160 patients, we compared perioperative outcomes of RAS and CLS while using the #Enzian classification to contextualize anatomical disease complexity.

The first key observation of our analysis is that operative time was not significantly different between the two approaches, although the median time was slightly higher in the RAS group. This finding is of particular relevance considering that the cases were significantly more complex in the RAS cohort and that multi-compartmental disease affecting the bladder (FB), the ureter (FU) and the diaphragm was almost exclusively observed in the RAS group. Restaino et al. have reported a trend towards longer operative times in robotic series of DE, with an average difference of 15 to 30 min compared to standard laparoscopy [9]. In a previous retrospective study and a meta-analysis, the authors reported a significantly longer operating time in robotic-assisted surgery for colorectal endometriosis [11, 12], while Raimondo et al. described a significant difference in operating room occupancy but no difference in operating time [13]. The time of operating room occupancy is of particular interest, as an improvement of workflow in operating room preparation, including the entire operating room team, can increase effectiveness in robotic-assisted surgery. Future research should pay particular attention to this important factor during implementation and processing of RAS programs.

The second relevant finding of our study is the significantly shorter postoperative length of hospital stay after RAS (median 5 vs. 6 days, *p* = 0.008). This is in line with the results by Crestani et al. [11], while other authors reported no differences in hospital stay when CLS and RAS were compared [12, 13]. In our study, the postoperative serum CRP level was significantly higher in the RAS group on days 2 and 3, which might be related to the substantially greater anatomical complexity and extent of resection in the RAS group. Elevated inflammatory markers were not associated with increased infectious morbidity, readmission or reoperation rates. The analyzed parameters in this study are not sufficiently robust as potential explanation for the faster recovery and thus shorter hospital stay after RAS. While a previous analysis of our group showed that approximately 70% of surgeons perceive RAS as advantageous in terms of patient outcome and satisfaction, reasons for this perception remain uncertain, as the usual parameters blood loss, complication rates and operative time are mostly similar when comparing RAS and CLS [6]. Thus, we hypothesize, that alternative factors, must be of importance and should be analyzed in future research. In our study, the significantly lower number of trocars in RAS (median 4 vs. 5, *p* = 0.004) might be one of these factors, possibly leading to a lower abdominal wall trauma. This positive effect could be increased through the very low number of instrument changes per RAS (6.4) in this cohort of complex endometriosis cases. The influence of lower intraabdominal pressure during RAS on patient outcomes has been analyzed in previous studies [14, 15], but was not addressed in our study. The combination of higher precision of RAS and the level of concentration of the surgeon working at the console might be another factor influencing patient outcomes. The eye-situs ratio during operative time in RAS compared to CLS could be analyzed in further research.

The third finding concerns reoperations. The reoperation rate was 1.7% (1/59) in the RAS group and 6.9% (7/101) in the CLS group (Fisher exact *p* = 0.26). With 160 patients and a low event rate the study is not adequately powered to detect a clinically meaningful difference of this magnitude. Numerically, the trend favors robotic-assisted surgery, which is noteworthy given the higher anatomical complexity of the cases managed with RAS. In the CLS group in six patients a postsurgical hematoma that required re-laparoscopy occurred, while this was not the case in the RAS group. The only conversion to open surgery occurred in the RAS group related to a major bleeding complication in a patient with a complex coagulation dysfunction that was previously unknown. Anastomotic/suture leakage was very rare in our cohort, occurring in 2 out of 160 patients (1.3%) and exclusively in the CLS group. This rate compares favorably with the literature, where leakage rates of 1.2–1.5% have been reported for nerve-sparing segmental resection for endometriosis [5]. No preventive stoma was performed in this cohort. In summary, our results are in line with previous publications, with no differences for complications, blood loss and re- hospitalization in both groups [16].

The fourth finding concerns the differentiation of #Enzian related anatomical complexity of endometriosis in all analyzed cases. Preoperative imaging-based assessment of disease extent with respective classification of all findings is crucial for treatment planning and case discussion in a multidisciplinary team conference and surgical planning including estimated operative time, choice of approach and instrument set-up, potential need for interdisciplinarity, risk stratification and adequate patient counseling [17, 6]. A standardized topographic classification is also essential for comparison of cases and interpretation of surgical outcomes, as the complexity of surgery and the risk for complications and thus perioperative morbidity in deep endometriosis surgery is influenced by disease extent in terms of anatomical localization and size of endometriosis lesions. Surgical outcomes in colorectal endometriosis do not only depend on the extent of bowel endometriosis itself and the chosen operative technique, but also on additional deep endometriosis lesions in the surrounding anatomical compartments including vagina, uterosacral ligaments and pelvic retroperitoneal vessels, nerves and ureters. The risk for nerve lesions might be higher in combined complex deep endometriosis scenarios, the rate for rectovagional fistula formation might be higher in patients with segmental rectal resection and vaginal exzision with suturing of the posterior vaginal wall and combined surgery including multiple organs like bowel, ureter, bladder or diaphragm carry a higher risk for complications [18]. Thus, #Enzian related differentiation of case complexity might allow for a more precise analysis of surgical outcomes.

In our center, the robotic platform was preferentially used for more complex cases with multicompartmental disease involving the bladder, the ureter and the diaphragm. The significant over-representation of FB (8.5% vs. 0.0%), FU (11.9% vs. 2.0%) and F-Diaphragm (10.2% vs. 0.0%) lesions in the RAS group represents a clear selection effect of using the robotic platform in the most demanding cases of complex deep endometriosis. In the reality of most centers the access to the robotic platform is limited and thus, a case selection is necessary. In our opinion, RAS should be used in complex multidisciplinary cases. At the same time, this selection effect supports the biological plausibility of the ergonomic advantages of the robotic platform in hard-to-reach anatomical regions such as the deep pelvis, the parametrium and the diaphragmatic dome. Maintaining comparable operative times and complication rates under these conditions underlines that the robotic platform may provide technical and ergonomical advantages particularly in anatomically demanding deep endometriosis surgery.

Our study is a retrospective, non-randomized single-center study with a possible selection bias in favor of the robotic platform in more complex cases. This selection effect limits the direct comparability of outcomes between the two groups and highlights the need for risk-adjusted or propensity-score-matched analyses in further studies. The use of the #Enzian classification, might provide a reproducible framework for such adjustments, although it is not yet a validated quantitative score. The relative weighting of #Enzian compartments in terms of surgical complexity remains unclear and the sole count of compartments involved might cause bias of interpretation.

Another important limitation is the sequential cohort design of our analysis. Surgical performance in the RAS cohort partially reflects previously acquired expertise in complex laparoscopic endometriosis surgery, whereas the converse transfer effect does not apply. Our observed findings might therefore be related to maturation of surgeon and multidisciplinary team rather than being a platform specific effect. The binary coding of the F-compartments (0/1) does not quantify the extent of the disease within each compartment. Long-term outcomes such as recurrence, quality of life, sexual function, defecation dysfunction and fertility were not systematically assessed in this analysis and will be the object of a dedicated follow-up study.

In summary, the present study shows that robotic-assisted surgery achieved perioperative outcomes and operative times comparable to conventional laparoscopy, but a significantly shorter hospitalization, despite a significantly higher anatomical complexity of the operated cases. Patients selected for RAS presented more extensive multicompartmental disease, including significantly higher rates of advanced rectal involvement, ureteral disease, bladder infiltration and diaphragmatic endometriosis.

## Conclusions

Robotic-assisted and conventional laparoscopic excision of colorectal endometriosis were safe and effective with comparable operative times and complication rates. Length of hospital stay was significantly shorter after RAS and reoperation rate numerically lower, although the complexity of robotic-assisted procedures was higher when considering the #Enzian related anatomical complexity of disease extent in all cases. Our findings support RAS as a safe minimally invasive approach for patients with complex multicompartmental colorectal endometriosis and highlight the possible importance of accounting for #Enzian-defined anatomical complexity when comparing surgical techniques. Prospective risk-adjusted studies are needed to confirm these observations.

## Data Availability

The analyzed data are available upon reasonable request to the first author of this publication.

## Abbreviations

RAS: Robotic-Assisted Surgery
CLS: Conventional Laparoscopic Surgery
DE: Deep Endometriosis
IQR: Interquartile Range
SD: Standard Deviation
CRP: C-Reactive Protein
LOS: Length of Stay
